# Supplementary material for: EGFP Reporters for Direct and Sensitive Detection of Mutagenic Bypass of DNA Lesions
Source: Biomolecules. 2020 Jun 13;10(6):902. doi: 10.3390/biom10060902 (PMC7357151; doi:10.3390/biom10060902)
Supplement: Supplementary file 1 [file biomolecules-10-00902-s001.pdf]

biomolecules

MDPI

EGFP reporters for direct and sensitive detection of mutagenic bypass of DNA lesions

Marta Rodriguez-Alvarez, Daria Kim and Andriy Khobta

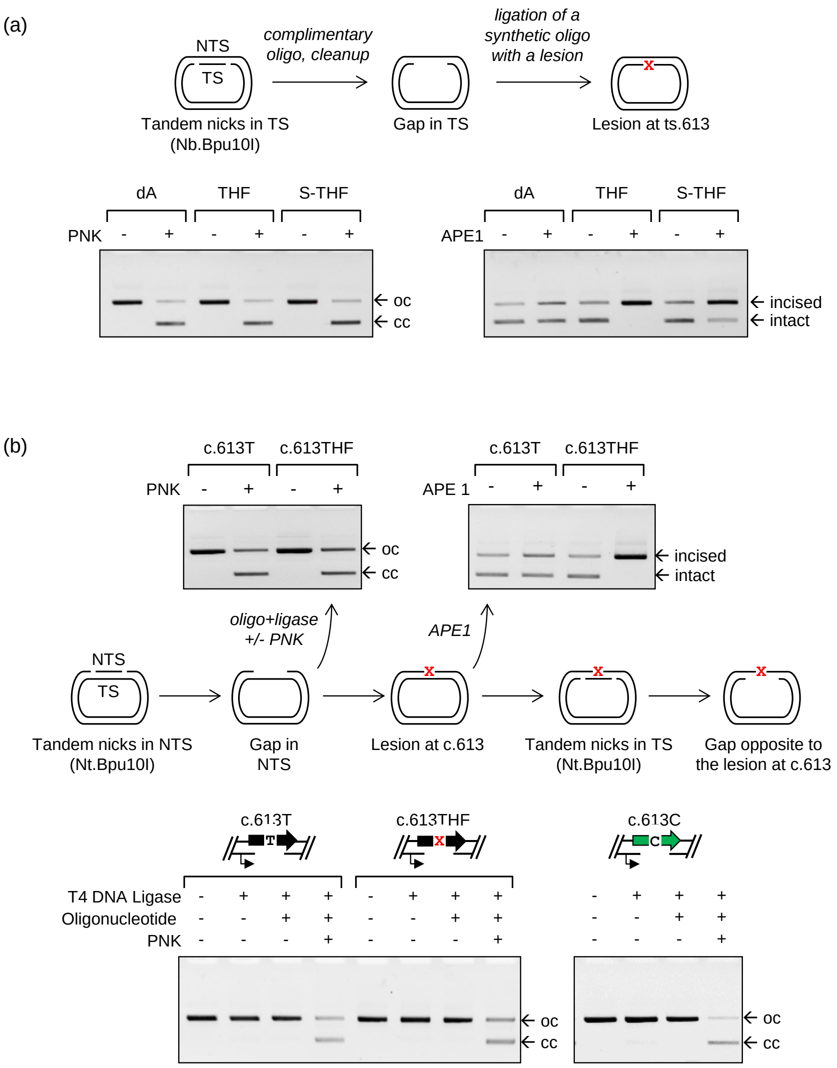

Supplementary Figure S1. Site-specific incorporation of AP lesions (THF or S-THF) into the pEGFP_Q205* to generate reporter constructs for detection of TM (a) and TLS (b). Positions of the lesions are indicated with "X": (a) Flow chart of incorporation of synthetic oligonucleotides containing dA/THF/S-THF into the transcribed strand (TS) of the gene and verification of the generated constructs. Incorporation of synthetic strands into the gap was confirmed by inhibition of the ligation reaction in the absence of polynucleotide kinase (PNK), as judged from the presence of the open circular (oc) and covalently closed (cc) forms of the vector DNA. The presence of THF AP lesion at the position ts.613 was verified by incision with human APE1, as described previously [30]. As expected, the incision at S-THF is inhibited by the phosphorothioate linkage. (b) Flow chart of preparation of constructs for the DNA TLS assay. Synthetic oligonucleotides containing dT or THF were incorporated into the gap generated in the non-transcribed DNA strand (NTS). Correct incorporation of synthetic DNA strands was confirmed by the ligation assay in the presence or absence of PNK. The presence of AP lesion was verified by the APE1 incision. Finally, the gaps were generated in the TS opposite to the incorporated dT/THF. Complete generation and correct size and position of the gaps in the TS was confirmed both by the inhibition of self-ligation of the obtained reporter constructs and by efficient ligation of the homologous oligonucleotide resulting in the cc-form in the presence of PNK (representative gel image below).

EGFP reporters for direct and sensitive detection of mutagenic bypass of DNA lesions

Marta Rodriguez-Alvarez, Daria Kim and Andriy Khobta

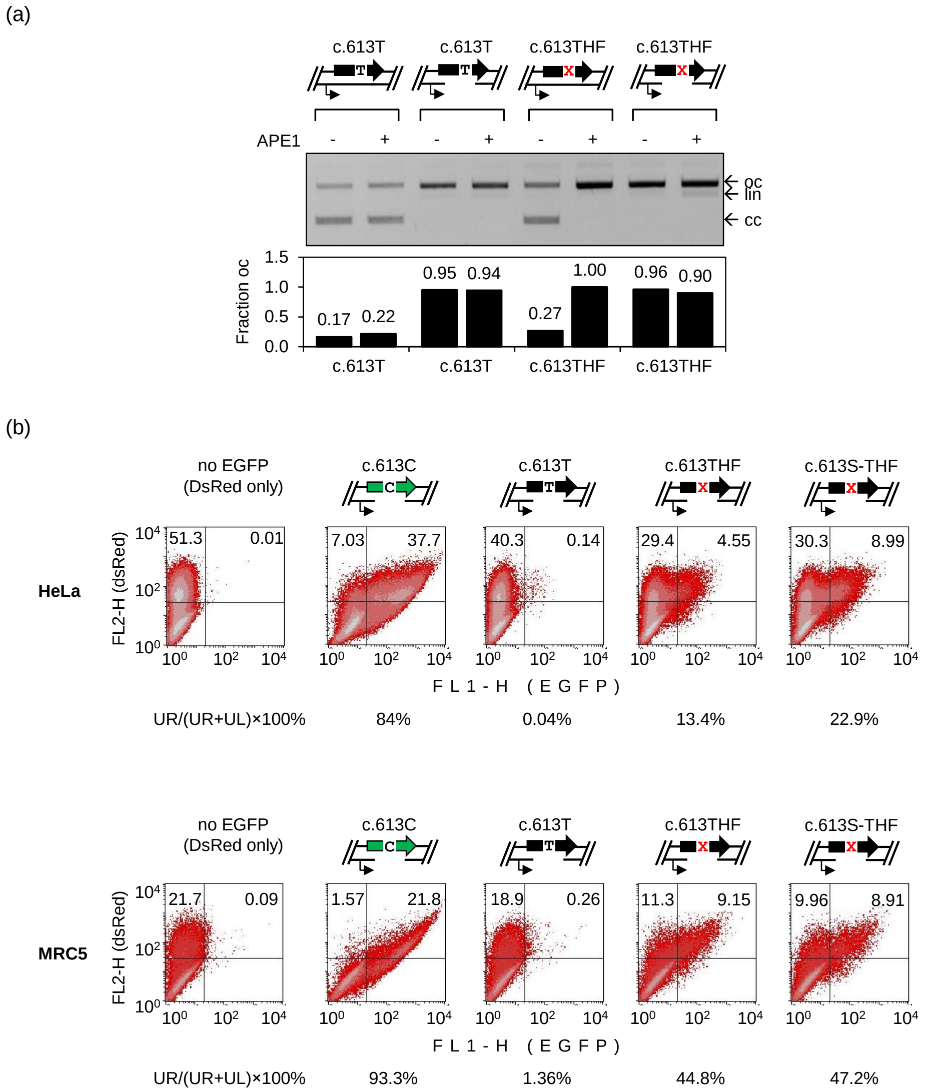

Supplementary Figure S2. Resistance of THF in the TLS template to APE1 and mutagenic TLS of synthetic AP lesions (THF and S-THF) in HeLa and MRC5 cell lines. (a) Quantitative analyses of APE1 activity towards the THF lesion in ssDNA and dsDNA. The respective constructs were incubated with APE1 (1 Unit/100 ng vector DNA). For THF in dsDNA, the incision was detected by conversion of the covalently closed DNA form (cc) into the open circular form (oc); for the lesion in ssDNA – by conversion of the open circular into the linear form (lin). Fractions of the open circular DNA were calculated based on densitometry of ethidium bromide-containing gels. The representative result demonstrates that THF is incised by APE1 far less efficiently in ssDNA than in dsDNA. (b) Detection of mutagenic TLS in HeLa and MRC5 cells transfected with constructs containing dT, THF and S-THF at the nucleotide 613 in the NTS and a gap in the opposite DNA strand. Reversion to fluorescent EGFP was analyzed by flow cytometry. Mutant frequencies were calculated as ratios of EGFP-positive cells (in the upper right quadrant, UR) to total transfected cell count (UR+UL). Representative results show detection of mutagenic TLS in both cell-lines and with both types of DNA lesions. The more stable S-THF lesion tended to yield more mutations than THF (especially in HeLa cells). Of note, MRC5 cells show a higher and more homogeneous gap repair efficiency, which may account for a more sensitive detection of mutations in this cell line.

EGFP reporters for direct and sensitive detection of mutagenic bypass of DNA lesions

Marta Rodriguez-Alvarez, Daria Kim and Andriy Khobta

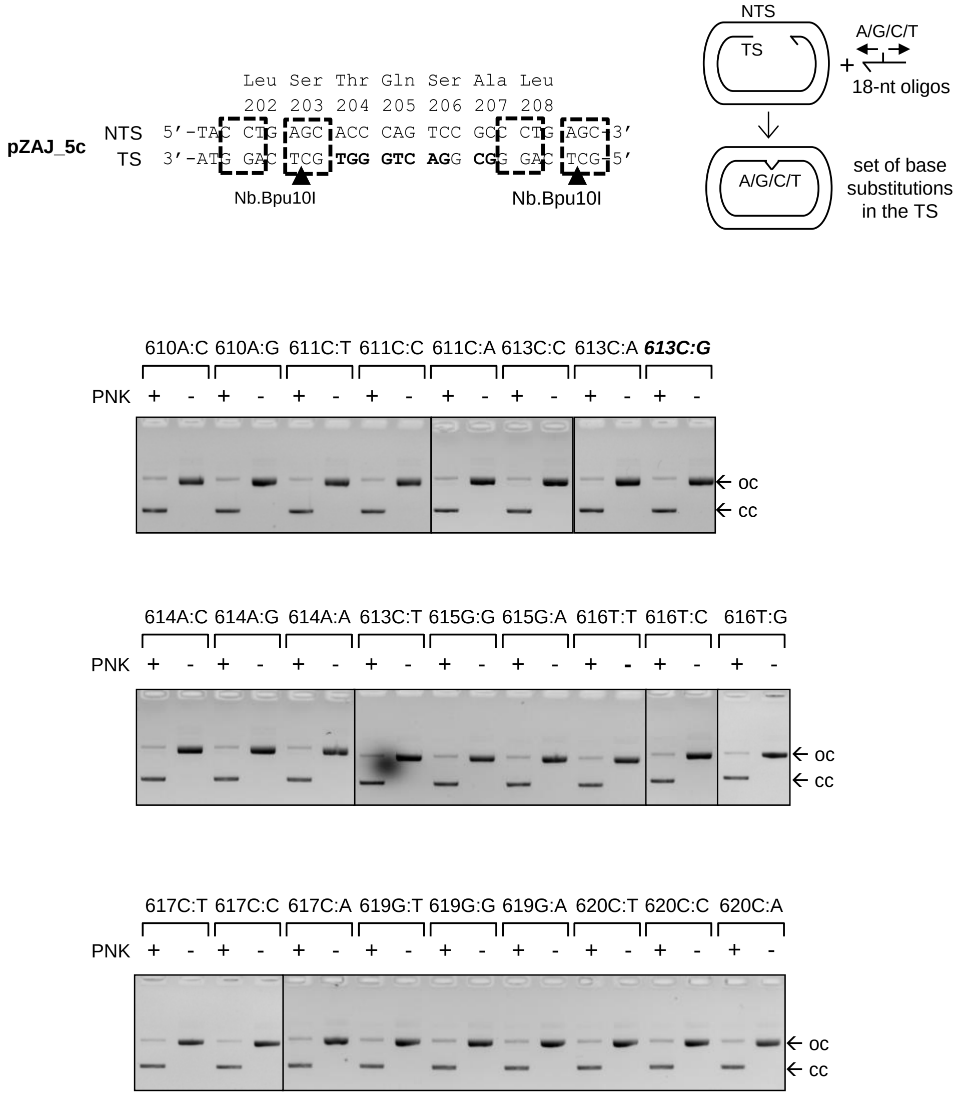

Supplementary Figure S3. Generation of EGFP expression constructs containing a set of 26 single nucleotide substitutions in the TS. Synthetic oligonucleotides (45-fold molar excess) containing varied bases at positions highlighted in bold type font were phosphorylated with polynucleotide kinase (PNK) and ligated into the 18-nucleotide single-strand gap generated in pZAJ_5C by nicking with Nb.Bpu10I at the available tandem sites and depletion of the excised native DNA strand (scheme above). Correct ligation of synthetic strands results in covalently closed (cc) form of the vector DNA, which migrates differently from the open circular (oc) form in the presence of ethidium bromide. Parallel ligations were performed in the absence of PNK to demonstrate that native DNA strand was completely replaced with synthetic strands in all constructs analysed. Detailed procedure for seamless incorporation of synthetic DNA strands containing mismatched nucleotides into circular vector DNA was described previously [32]. Labels above the lanes indicate the nature of mismatches (NTS:TS) generated at the indicated nucleotide positions. The ***613C:G*** construct contains fully matched synthetic DNA strand, which restores the original C:G base pair. This construct was used as a reference for fully functional EGFP in transfection experiments.
